# Hypocaloric Diet Prevents the Decrease in FGF21 Elicited by High Phosphorus Intake

**DOI:** 10.3390/nu10101496

**Published:** 2018-10-13

**Authors:** Carmen Pineda, Rafael Rios, Ana I. Raya, Mariano Rodriguez, Escolastico Aguilera-Tejero, Ignacio Lopez

**Affiliations:** 1 Department Medicina y Cirugia Animal, University of Cordoba, 14071 Cordoba, Spain; v32pimac@uco.es (C.P.); rafariosvaro@me.com (R.R.); v82rabea@uco.es (A.I.R.); l02lovii@uco.es (I.L.); 2 Maimonides Biomedical Research Institute of Cordoba (IMIBIC), Reina Sofia University Hospital, University of Cordoba, 14004 Cordoba, Spain; marianorodriguezportillo@gmail.com

**Keywords:** fibroblast growth factor 21, phosphorus, calories, rat

## Abstract

The effect of dietary phosphorus (P) on fibroblast growth factor 21 (FGF21)/β-klotho axis was investigated in rats that were fed diets with: Normal (NP) or high P (HP) and either normal (NC), high (HC) or low calories (LC). Sampling was performed at 1, 4 and 7 months. Plasma FGF21 concentrations were higher (*p* < 0.05) in NC and HC than in LC groups. Increasing P intake had differing effects on plasma FGF21 in rats fed NC and HC vs. rats fed LC at the three sampling times. When compared with the NP groups, FGF21 concentrations decreased at the three sampling points in rats fed NC-HP (80 vs. 194, 185 vs. 382, 145 vs. 403 pg/mL) and HC-HP (90 vs. 190, 173 vs. 353, 94 vs. 434 pg/mL). However, FGF21 did not decrease in rats fed LC-HP (34 vs. 20, 332 vs. 164 and 155 vs. 81 pg/mL). In addition, LC groups had a much lower liver FGF21 messenger ribonucleic acid/glyceraldehyde 3-phosphate dehydrogenase (mRNA/GAPDH) ratio (0.51 ± 0.08 and 0.56 ± 0.07) than the NC-NP (0.97 ± 0.14) and HC-NP (0.97 ± 0.22) groups. Increasing P intake reduced liver FGF21 mRNA/GAPDH in rats fed NC and HC to 0.42 ± 0.05 and 0.37 ± 0.04. Liver β-klotho mRNA/GAPDH ratio was lower (*p* < 0.05) in LC groups (0.66 ± 0.06 and 0.59 ± 0.10) than in NC (1.09 ± 0.17 and 1.03 ± 0.14) and HC (1.19 ± 0.12 and 1.34 ± 0.19) groups. A reduction (*p* < 0.05) in β-klotho protein/α-tubulin ratio was also observed in LC groups (0.65 ± 0.05 and 0.49 ± 0.08) when compared with NC (1.12 ± 0.11 and 0.91 ± 0.11) and HC (0.93 ± 0.17 and 0.87 ± 0.09) groups. In conclusion β-klotho is potently regulated by caloric restriction but not by increasing P intake while FGF21 is regulated by both caloric restriction and increased P intake. Moreover, increased P intake has a differential effect on FGF21 in calorie repleted and calorie depleted rats.

## 1. Introduction

A growing body of evidence is being gathered about the relationship between energy metabolism and mineral metabolism. This association includes links of obesity with osteoporosis [1], calcium intake [2] and hyperparathyroidism [3]. Moreover, obesity is related to vitamin D since vitamin D-receptor knockout rodents display a lean phenotype [4] and obese people tend to have low vitamin D levels [5]. Leptin, an adipokine that is elevated in obese individuals, has been shown to stimulate fibroblast growth factor 23 (FGF23) [6] and parathyroid hormone secretion [7]. Furthermore, mineral metabolism is also interrelated with glucose homeostasis—e.g., magnesium and osteocalcin have protective actions against diabetes and metabolic syndrome [8,9].

In addition to its skeletal functions, phosphorus (P) is closely related to energy metabolism—phosphoproteins are involved in mitochondrial oxidative phosphorylation and energy for essential metabolic processes is stored in high energy phosphate bonds of adenosine triphosphate [10]. Thus, adequate nutritional intake of P is essential for healthy life. However, the deleterious consequences of increased P intake are also well known, especially in patients with reduced renal function [11]. Western diets are typically rich in P and the use of P as a food additive further increases the P content of many processed foods. Moreover, the P used as an additive is inorganic P which is much more readily absorbed in the intestine than the P naturally contained in foods [12].

Within the superfamily of fibroblast growth factors (FGFs), two hormone-like molecules are key regulators of energy metabolism (FGF21) and mineral metabolism (FGF23). Fibroblast growth factor 21, that is mainly synthesized in the liver, stimulates glucose uptake by adipocytes, increases energy expenditure and improves the lipid profile [13]. Fibroblast growth factor 23, which is produced by bone cells, participates in mineral metabolism as a major phosphaturic hormone [14]. Both FGF21 and FGF23 need a co-factor to interact with their receptors. These cofactors are two distinct molecules: β-klotho for FGF21 and α-klotho for FGF23.

Although the main actions of FGF23 are related to P homeostasis, there is evidence of a regulation of FGF23 by energy metabolism. In addition to being regulated by leptin [6], an increase in circulating levels of FGF23 has been reported in rats fed high fat diets [15,16]. The mechanism for increased FGF23 production after eating energy-dense diets seems to be related to decreased expression of renal α-klotho: α-klotho down-regulation generates FGF23 resistance and thus more FGF23 is needed for P excretion [15].

The role of the FGF21/β-klotho axis in mineral metabolism is less clear. Based on a recent study that has shown that the tubular load of P modulates the expression of α-klotho in the kidney [17], we hypothesize that increasing hepatic P load by feeding a diet with high P content would regulate the FGF21/β-klotho axis. Since both fasting, and the caloric content of the diet, are known to influence β-klotho and FGF21, the effect of a high P diet was explored in rats fed normo-, hyper- and hypo-caloric diets.

## 2. Materials and Methods

### 2.1. Ethics

All experimental protocols were reviewed and approved by the Ethics Committee for Animal Research of the University of Cordoba and by Junta de Andalucia (Spain) (Ethical Code Number 30/10/2017/148, date 8 November 2017). All protocols were carried out in accordance with the approved guidelines. They followed the guiding principle laid down by the Higher Council of Scientific Research of Spain following the normal procedures directing animal welfare and adhered to the recommendations included in the Guide for Care and Use of Laboratory Animals (US Department of Health and Human Services, NIH) and European laws (Art. 41.1, Real Decreto 53/2013, 01/02 Dec 2012/707/UE) and regulations on protection of animals, under the advice of specialized personnel.

### 2.2. Animals and Diets

Two month-old Wistar rats, provided by the Animal Housing Facilities of the University of Cordoba (Cordoba, Spain), were housed with a 12 h/12 h light/dark cycle. Appropriate measures were taken to ensure animal welfare and to address the basic behavioral and physiological needs of rats.

Diets with two P concentrations were used in the experiments: Normal P (0.6%) diet (NP) and high P (1.2%) diet (HP). Independent of their P content, diets had a normal energy content (NC diet) providing metabolizable energy = 3528 kcal/kg (Altromin C1090-10, Altromin Spezialfutter GmbH, Lage, Germany), a high energy content (HC diet) providing metabolizable energy = 5241 kcal/kg (Altromin C 1090-60, Altromin Spezialfutter GmbH, Lage, Germany), or a low energy content (LC diet) providing metabolizable energy = 1314 kcal/kg (Altromin C1012, Altromin Spezialfutter GmbH, Lage, Germany). All diets contained 0.6% of Ca and 500 IU/g of vitamin D.

### 2.3. Experimental Design

Rats were allotted to 6 experimental groups (*n* = 8). Rats in group 1 were fed the NC-NP diet, rats in group 2 were fed the NC-HP diet, rats in group 3 were fed the HC-NP diet, rats in group 4 were fed the HC-HP diet, rats in group 5 were fed the LC-NP diet and rats in group 6 were fed the LC-HP diet. Diets were fed ad libitum for 7 months. Food intake and body weight were measured every week. Blood samples were obtained between 09.00 and 11.00 h from fasted (12 h) rats after the animals had been receiving the experimental diets for 1, 4 and 7 months. Blood sampling at 1 and 4 months was performed on anesthetized (inhaled sevofluorane) rats and blood was obtained from the jugular vein. Blood samples at 7 months were obtained from the abdominal aorta, at the time of sacrifice. At the end of the experiment (7 months), rats were sacrificed by exsanguination under general anesthesia (inhaled sevoflurane). In addition to blood, liver samples were also obtained, frozen at −80 °C and stored until processing for ribonucleic acid (RNA) and protein extraction.

### 2.4. Blood Chemistries

After blood collection, glucose was determined using a blood glucose meter (Bayer Consumer Care AG, Basel, Switzerland). Then, plasma was separated by centrifugation and stored at –20 °C until assayed. Plasma total cholesterol, triglycerides and P were measured by spectrophotometry (BioSystems SA, Barcelona, Spain). ELISA tests were used to quantify plasma FGF21 (EMD Millipore Corporation, St. Charles, MO, USA), leptin and adiponectin (EMD Millipore Corporation, St. Charles, MO, USA).

### 2.5. RNA Extraction and Quantitative Real-Time PCR(RT-PCR)

Study of liver β-klotho and FGF21 mRNA was performed by Quantitative Real-Time PCR (RT-PCR). Liver tissue was disrupted using liquid nitrogen and grinded thoroughly with a mortar. Total RNA was extracted using the chloroform and isopropanol precipitation method and a treatment with deoxyribonuclease I (DNAse I) Amplification Grade (Sigma-Aldrich, St. Louis, MO, USA). Fifty nanograms of total RNA was used to analyze messenger ribonucleic acid (mRNA) expression in the LightCycler thermal cycler system (Roche Diagnostics, Indianapolis, IN, USA). RT-PCR was performed in one step using the QuantiTect SYBR Green RT-PCR kit (Qiagen GmbH, Hilden, Germany) following the manufacturer´s protocol. Primers for β-klotho were designed with the free Oligo 7 software. The FGF21 primer was used based on a previous publication [18]. The expression of target genes was normalized to glyceraldehyde 3-phosphate dehydrogenase (GAPDH) as housekeeping and calculated according to the 2∆ (∆ CT) method.

Primer sequences are listed below:

β-klotho: Forward primer: 5′-TCAACCAGGTTCTTCAAGCAATA-3′; Reverse primer: 5′-GGTTTCCTCTCTTTCTGCTCAC-3′.

FGF21: Forward primer: 5′-CAAATCCTGGGTGTCAAAGC-3′; Reverse primer: 5′-GCCTCAGACTGGTACACATTG-3′.

GAPDH: Forward primer: 5′-AGGGCTGCCTTCTCTTGTGAC-3′; Reverse primer: 5′-TGGGTAGAATCATACTGGAACATGTAG-3′.

### 2.6. Protein Extraction and Western Blot

Proteins were isolated from liver tissue by using a lysis buffer containing 4-(*2-*hydroxyethyl)-1-piperazineethanesulfonic acid (HEPES) (10 mmol/L), potassium chloride (KCl) (10 mmol/L), ethylenediaminetetraacetic acid (EDTA) (0.1 mmol/L), ethylene glycol tetraacetic acid (EGTA) (0.1 mmol/L), dithiothreitol (DTT) (1mmol/L), phenylmethylsulfonyl fluoride (PMSF) (0.5 mmol/L), protease inhibitor cocktail (70 µg/mL), and I-Gepal CA-630 (0.6%), pH 7.9 (Sigma Aldrich, St. Louis, MO, USA). Protein concentration was determined by the Bradford method. For Western blot analysis, 50 µg of protein was electrophoresed on a 10% SDS-polyacrilamide gel (Invitrogen, Carlsbad, CA, USA) and electrophoretically transferred (Transfer Systems, BioRad, Hercules, CA, USA) from the gels onto nitrocellulose membranes (Invitrogen, Carlsbad, CA, USA). The following steps were performed with gentle shaking. Membranes were incubated in TBST (Tris-buffered saline tween 20) solution (20 mM Tris-HCl (pH 7.6), 0.2% Tween 20, 150 mM NaCl) (Sigma Aldrich, St. Louis, MO, USA), and 5% nonfat dry milk (Bio-Rad, Hercules, CA, USA) at room temperature for 1.5h to avoid nonspecific binding. Membranes were then washed with TBST buffer (the same composition as TBST without nonfat dry milk) and incubated overnight at 4 °C with a rabbit anti-β-klotho antibody (LS-B3568, LifeSpan BioSciences, Inc., Seattle, WA; 1 µg/mL). The membranes were then washed with TBST buffer and immunolabeled using a peroxidase-conjugated secondary antibody (1:5000 dilution; Santa Cruz Biotechnology Inc., Santa Cruz, CA, USA). Finally, they were revealed on autoradiographic film using ECL Plus Western Blotting Detection System (GE Healthcare, Piscataway, NJ, USA). Alpha tubulin (Abcam, Cambridge, UK) was used as a housekeeping protein to ensure equal loading of the gels. Protein levels were quantified using ImageJ software (National Institutes of Health, Bethesda, MD, USA).

### 2.7. Statistical Analysis

Values are expressed as the mean ± standard error (SE). The difference between means for two different groups was determined by *t*-test; the difference between means for three or more groups was assessed by ANOVA. The Fisher LSD test was used as a post-hoc procedure. *p* < 0.05 was considered significant.

## 3. Results

### 3.1. Energy Intake and Body Weight

**Figure 1:**
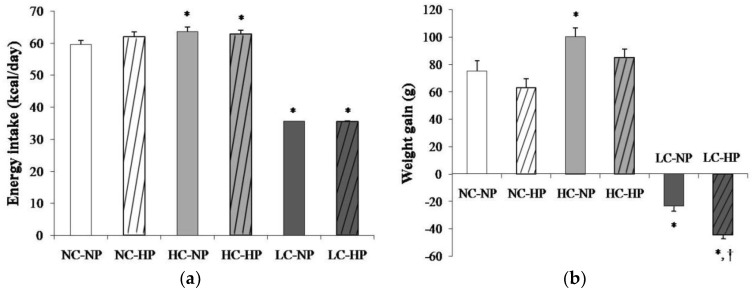
(**a**) Energy intake and (**b**) weight gain after the 7 months that the experiments lasted in rats fed diets with normal calorie-normal phosphorus (NC-NP), normal calorie-high phosphorus (NC-HP), high calorie-normal phosphorus (HC-NP), high calorie-high phosphorus (HC-HP), low calorie-normal phosphorus (LC-NP) and low calorie-high phosphorus (LC-HP). *n* = 8 rats per group. *p < 0.05 vs. NC-NP, ^†^ *p* < 0.05 vs. LC-NP.

Mean energy intake was 59.6 ± 1.2 kcal/day in rats fed a NC-NP diet. Energy intake was slightly increased (*p* < 0.01) in rats fed HC-NP, 63.6 ± 1.3 kcal/day, and markedly decreased (*p* < 0.0001) in rats fed LC-NP, 35.1 ± 0.1 kcal/day. The phosphorus content of the diet did not influence energy intake (Figure 1a). At the beginning of the study all rats had similar body weight that ranged between 239 and 251 g. During the 7 months that the experiment lasted, rats fed NC and HC diets experienced an increase in body weight that tended to be higher in rats fed normal P. Rats fed HC gained more weight than rats fed NC, but the differences were only significant in the NP groups. A progressive decrease in body weight was observed in rats fed LC diets. By the end of the experiment rats on the LC-NP diet had lost 23.4 ± 3.7 g. The decrease in body weight was more accentuated in rats fed the LC-HP diet, 44.3 ± 3.2 g, *p* = 0.001 vs. LC-NP (Figure 1b).

### 3.2. Biochemical Data

**Figure 2:**
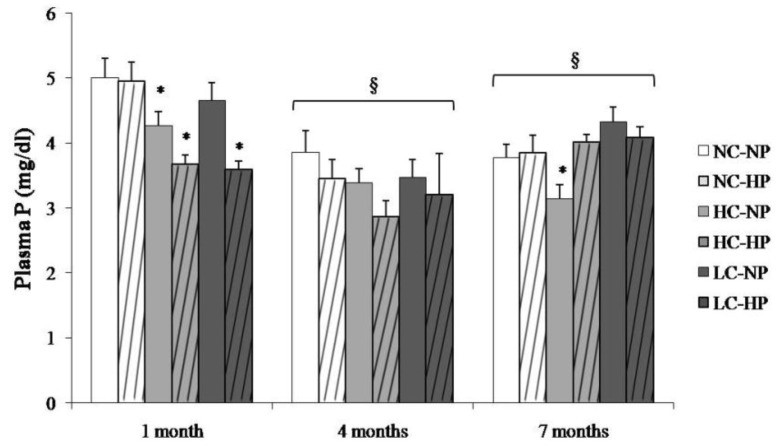
Plasma concentrations of phosphorus (P) at the three sampling times (1, 4 and 7 months) in rats fed diets with normal calorie-normal phosphorus (NC-NP), normal calorie-high phosphorus (NC-HP), high calorie-normal phosphorus (HC-NP), high calorie-high phosphorus (HC-HP), low calorie-normal phosphorus (LC-NP) and low calorie-high phosphorus (LC-HP). *n* = 8 rats per group. * *p* < 0.05 vs. NC-NP. ^§^ *p* < 0.05 vs. 1 month.

Plasma P concentrations in the study groups at the three sampling times are depicted in Figure 2. Plasma P was higher at 1 month and then stabilized at 4 and 7 months. The caloric content of the diets did not influence plasma P concentration. Plasma P was not increased in the HP groups, in fact, a decrease in plasma P was identified in the HC-HP and LC-HP groups at 1 month (Figure 2).

Plasma triglycerides were lower in the LC-NP group (22.3 ± 1.7 mg/dL) and higher in the HC-NP group (83.1 ± 11.4 mg/dL) than in NC-NP group (54.8 ± 11.1 mg/dL). High P intake decreased plasma triglycerides in the HC-HP group to 39.6 ± 3.4 mg/dL (*p* < 0.0001). Plasma cholesterol concentrations, which tended to decrease with low caloric intake, increased significantly (*p* < 0.05) in LC-HP rats when compared with LC-NP rats. Diets with HP significantly decreased plasma glucose both in NC (94.1 ± 4.4 vs. 116.9 ± 6.1 mg/dL, *p* < 0.05) and LC (74.2 ± 5.7 vs. 109.0 ± 19.6 mg/dL, *p* < 0.01) rats. Plasma leptin concentrations were lower in LC groups (0.6 ± 0.2 and 0.6 ± 0.1 ng/mL), intermediate in NC groups (4.2 ± 0.5 and 3.1 ± 0.2 ng/mL) and slightly higher in HC groups (5.3 ± 0.8 and 4.1 ± 0.5 ng/mL). When compared with NC, adiponectin tended to be lower in rats fed HC and higher in rats fed LC. Rats fed HP tended to have lower leptin and higher adiponectin concentrations, and differences were significant for adiponectin in LC groups (16.6 ± 2.3 vs. 10.5 ± 0.6 ng/mL, *p* < 0.01) (Table 1).

**Figure 3:**
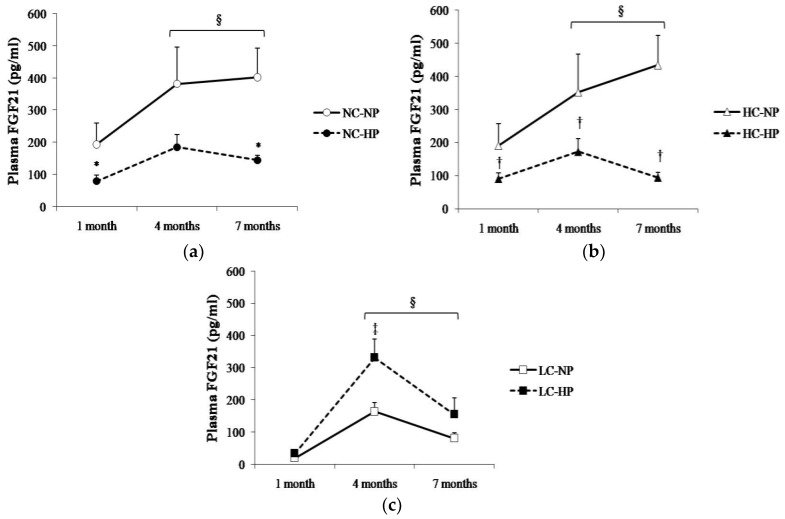
Plasma concentrations of fibroblast growth factor 21 (FGF21) at the three sampling times (1, 4 and 7 months) in rats fed diets with (**a**) normal calorie-normal phosphorus (NC-NP) and normal calorie-high phosphorus (NC-HP); (**b**) high calorie-normal phosphorus (HC-NP) and high calorie-high phosphorus (HC-HP); (**c**) low calorie-normal phosphorus (LC-NP) and low calorie-high phosphorus (LC-HP). *n* = 8 rats per group. * *p* < 0.05 vs. NC-NP, ^†^ *p* < 0.05 vs. HC-NP, ^‡^ *p* < 0.05 vs. LC-NP. ^§^ *p* < 0.05 vs. 1 month.

Plasma FGF21 changed over time following a similar pattern in the six experimental groups, with the higher values being recorded between 4–7 months. Plasma FGF21 concentrations were consistently higher (*p* < 0.05) in the groups that received NC and HC than in the groups that received LC. Thus, rats in NC-NP and HC-NP groups had mean FGF21 concentrations that ranged between 190 pg/mL, at 1 month, and 434 pg/mL, at 7 months. By contrast, mean values of FGF21 in rats fed LC-NP diet were much lower: 20 pg/mL at 1 month, and 81 pg/mL at 7 months. Interestingly, increasing the P content of the diet had differing effects on plasma FGF21 in rats fed NC and HC vs. rats fed LC. When compared with the NP groups, FGF21 concentrations decreased at the three sampling points in rats fed NC-HP (80, 185, 145 pg/mL, *p* < 0.05 at 1 and 7 months) and HC-HP (90, 173, 94 pg/mL, *p* < 0.05 at 1, 4 and 7 months). However, FGF21 did not decrease in rats fed LC-HP, in fact, FGF21 tended to increase (34, 332 and 155 pg/mL) and the differences were significant (*p* = 0.007) at 4 months, when compared with the LC-NP group (Figure 3).

### 3.3. RT-PCR and Western Blot Results

**Figure 4:**
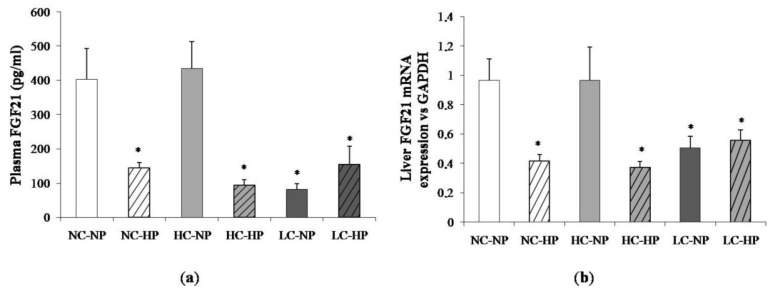
(**a**) Plasma fibroblast growth factor 21 (FGF21) concentrations and (**b**) liver mRNA expression of FGF21 at the end of the experiments (7 months) in rats fed diets with normal calorie-normal phosphorus (NC-NP), normal calorie-high phosphorus (NC-HP), high calorie-normal phosphorus (HC-NP), high calorie-high phosphorus (HC-HP), low calorie-normal phosphorus (LC-NP) and low calorie-high phosphorus (LC-HP). GAPDH: glyceraldehyde 3-phosphate dehydrogenase. *n* = 8 rats per group. * *p* < 0.05 vs. NC-NP.

Figure 4 shows plasma concentrations of FGF21 and liver mRNA expression of FGF21 at the end of the experiments (7 months). Like in plasma, the liver FGF21 mRNA/GAPDH ratio was much lower in the LC-NP group (0.51 ± 0.08) than in the NC-NP (0.97 ± 0.14) and HC-NP (0.97 ± 0.22) groups (*p* = 0.01). Increasing the P intake reduced liver FGF21 mRNA/GAPDH in rats fed normal calories (NC-HP, 0.42 ± 0.05, *p* = 0.003 vs. NC-NP) and high calories (HC-HP, 0.37 ± 0.04, *p* = 0.001 vs. HC-NP) to levels comparable to the LC groups. No significant differences were found between LC-NP and LC-HP groups (0.51 ± 0.08 and 0.56 ± 0.07).

**Figure 5:**
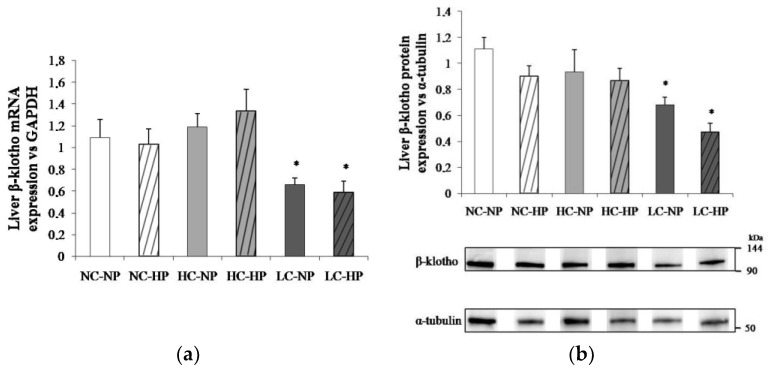
Liver β-klotho expression ((**a**) mRNA; (**b**) protein) at the end of the experiments (7 months) in rats fed diets with normal calorie-normal phosphorus (NC-NP), normal calorie-high phosphorus (NC-HP), high calorie-normal phosphorus (HC-NP), high calorie-high phosphorus (HC-HP), low calorie-normal phosphorus (LC-NP) and low calorie-high phosphorus (LC-HP). GAPDH: glyceraldehyde 3-phosphate dehydrogenase. *n* = 8 rats per group. *p < 0.05 vs. NC-NP.

The caloric content of the diets regulated liver β-klotho both at transcriptional and translational levels. Thus, liver β-klotho mRNA/GAPDH ratios were much lower (*p* < 0.01) in the LC groups (0.66 ± 0.06 and 0.59 ± 0.10) than in the NC groups (1.09 ± 0.17 and 1.03 ± 0.14) and the HC groups (1.19 ± 0.12 and 1.34 ± 0.19). A similar reduction (*p* < 0.05) in β-klotho/α-tubulin ratio was observed in the LC groups (0.65 ± 0.05 and 0.49 ± 0.08) when compared with the NC groups (1.12 ± 0.11 and 0.91 ± 0.11) and the HC groups (0.93 ± 0.17 and 0.87 ± 0.09). Increasing P intake did not affect either β-klotho mRNA or protein in any (NC, HC and LC) of the diets (Figure 5).

## 4. Discussion

This study was designed to test the hypothesis that increasing the P content of the diet would reduce β-klotho expression and subsequently lead to an increase in FGF21 synthesis and secretion. Our results, which are somewhat unexpected, show that β-klotho is preferentially regulated by caloric intake rather than by P intake. Increasing P intake resulted in a differential response in FGF21 that decreased, both in liver and in blood, in rats fed NC and HC diets but not in rats fed LC diets.

The influence of P on glucose and lipid metabolism has been known for a long time. In humans, hypophosphatemia has been associated to glucose intolerance due to tissue insensitivity to insulin [19] and glucose disposal rate has been reported to increase after phosphate infusion [20]. Experiments in rats have shown that dietary P deprivation stimulates liver gluconeogenesis and glucogenolysis suggesting that the liver may be implicated in the modulation of glucose homeostasis induced by P deficiency [21,22]. In mice, dietary P restriction has been reported to induce lipid accumulation in the liver through dysregulation of genes involved in the hepatic metabolism of cholesterol [23]. More recently, an elegant study by Abuduli et al. [24] has described with detail the influence of dietary phosphate on glucose and lipid metabolism, demonstrating an effect not only of P restriction but also of increased P intake. Their data show that a high P diet improves glucose regulation, down-regulates hepatic lipid synthesis and increases the expression of proteins that prevent visceral fat accumulation. In agreement with Abuduli et al. [24] we have also found that increasing the P content of the diet results in a tendency of decreased body weight, lower plasma glucose and improved triglycerides and adipokine profile (decrease in leptin and increase in adiponectin). In addition, our data demonstrate that the effects of increased P intake on glucose and lipid metabolism are not restricted to calorie-repleted rats but are also evident in calorie-depleted rats.

The relationship between dietary P and energy metabolism is bidirectional. As explained above, P can regulate energy metabolism, but it is also known that energy intake modulates parameters related to P regulation. Leptin, an adipokine that has a profound influence on the regulation of energy intake and that is consistently increased in obesity [25], has been shown to regulate the synthesis and secretion of FGF23, a major phosphaturic hormone [6]. Recent studies have shown that both high-fat [15] and high P [17] intake regulate the FGF23/α-klotho axis by decreasing the expression of renal α-klotho and increasing the synthesis and secretion of FGF23. Based on this data, we hypothesized that hepatic P overload secondary to high P intake would regulate the FGF21/β-klotho axis and that the regulatory effects would be different in energy-repleted vs. energy-depleted rats.

This study was designed to evaluate the influence of high P intake on rats fed diets with three levels of energy content. However, the difference in energy intake between the rats in the NC and HC groups, while statistically significant, was not substantial and therefore the conclusions that can be obtained from rats fed HC are limited. As it has been previously reported by us and others [15,26] when being offered hypercaloric diets, rats tend to reduce food intake. As a consequence, although the metabolizable energy of HC and NC diets was considerably different (5241 vs. 3528 kcal/kg), and daily caloric intake was only slightly higher in HC rats than in NC rats. Even though in the course of the 7 months of the experiment rats fed HC showed increased body weight and other biochemical signs of obesity/metabolic syndrome (e.g., hypertriglyceridemia and hyperleptinemia), the results obtained in the HC group were not very different from the NC group. However, rats in the LC groups showed marked differences in energy intake to the other two groups (NC and HC) and this allowed a better evaluation of the effect of energy depletion on the parameters under study.

To our knowledge, there are no previous data about the influence of a calorie deprived diet on liver β-klotho expression but there are several studies on the effect of obesity/high energy intake on liver β-klotho. Hepatic β-klotho has been shown to be elevated in obese human subjects, and in diet-induced obese mice [27,28]. However, Fletcher et al. [29] found lower levels of liver β-klotho in obese hyperphagic Otsuka Long-Evans Tokushima fatty (OLETF) rats than in control Long-Evans Tokushima Otsuka (LETO) rats. Our finding of a reduction in β-klotho expression in rats eating hypocaloric diets is consistent with the data that point towards a negative regulation of hepatic β-klotho by low energy intake. Rats fed high P diets showed a tendency of decreased β-klotho, but the differences with the animals fed normal P were small and non-significant. Therefore, high P intake does not seem to be a potent regulator of hepatic β-klotho.

Conversely, FGF21 was clearly regulated by both caloric intake and P intake. The effect of caloric intake on FGF21 is rather controversial and a variety of differing results have been published. Circulating levels of FGF21 are consistently elevated in obese individuals due to increased hepatic synthesis rather than to FGF21 resistance [30]. On the other hand, fasting is also known to upregulate FGF21, which has been defined as a central molecule in the physiologic response to starvation [31]. More recent data indicate that protein restriction rather than calorie restriction is responsible for the increase in FGF21 in response to starvation [32]. In ApoE-deficient mice subjected to long-term caloric restriction, FGF21 decreased when compared with controls during the first 25 weeks of caloric restriction and then increased over the controls from 30 to 64 weeks [33]. Plasma FGF21 has also been reported to be upregulated in mice subjected to caloric restriction for 4–6.5 weeks [34]. In our study, plasma FGF21 concentrations were consistently lower at the three sampling times in rats fed hypocaloric diets than in rats fed normal and high calories. Several factors may explain the discrepancy with studies that have found an increase in FGF21 after calorie restriction: (a) Previous studies have been conducted in mice which may behave differently to rats, (b) time of sampling may be important because FGF21 has been shown to be affected by circadian rhythm [35] and in some studies, e.g., Thompson et al. [34], mice were sampled at night time, while in our study the rats were sampled in the morning, between 9.00 and 11.00 h. In our opinion, the most important factor to explain the FGF21 concentrations detected in rats fed LC is the fact that these rats were subjected to calorie restriction but not to protein restriction, which seems the main trigger of FGF21 elevation [32]. In fact, the LC diet used in the present study has a high protein (52%) content. This is in contrast with most studies in which caloric restriction was achieved by reducing the amount of normocaloric food supplied to the animals, which means that they also ingested less proteins.

The main objective of this work was not to study the influence of caloric restriction but rather to evaluate the influence of high P intake on FGF21. High P intake resulted in a consistent decrease in plasma FGF21 in rats fed NC and HC but not in rats fed LC. Very little data relating P intake and plasma FGF21 can be found in the literature. Chun et al. [36] reported that rats fed 1.2% P had higher FGF21 plasma concentrations than rats fed 0.2% P, although the differences were not significant. In a study designed to investigate the effect of P restriction on FGF15, Nakahashi et al. [37] reported an increase in FGF21 mRNA levels in mice fed 1.2% P when compared with mice fed 0.6%; however, the 1.2% values were not different from those obtained in mice fed 0.2% P, 0.1% P and 0.02% P. Thus, although poorly documented, the sparse data in the literature points toward an increase in FGF21 after high P intake. Our data confirms this trend, but only in rats fed low calorie diets. In calorie repleted rats, increased P intake consistently decreased FGF21. It is important to stress that these results are quite robust since they were confirmed by sampling the same animals three times over a period of 7 months. The reasons for the different FGF21 response to HP in rats fed normal or high and low calories are not clear. In calorie repleted rats, the effect of high P intake on energy metabolism (improved glucose regulation, down-regulation of hepatic lipid synthesis and prevention of visceral fat accumulation), is likely to deem FGF21 less necessary, thus, explaining the reduction in circulating FGF21. However, rats fed low calories that already have low levels of FGF21 and β-klotho, probably cannot afford a further down-regulation of the FGF21/β-klotho axis. Actually, it seems that high P intake has similar metabolic consequences as caloric deprivation but that their effects are not additive.

## 5. Conclusions

The results of this study show that liver β-klotho is potently regulated by reduced caloric intake but not by increasing P intake, while FGF21 (hepatic and plasmatic) is regulated by both caloric deficit and increased P intake. Moreover, increased P intake decreases FGF21 in calorie repleted rats but not in calorie depleted rats.

## Figures and Tables

**Table 1:** Blood biochemistry at the end of the experiments (7 months) in rats fed diets with normal calorie-normal phosphorus (NC-NP), normal calorie-high phosphorus (NC-HP), high calorie-normal phosphorus (HC-NP), high calorie-high phosphorus (HC-HP), low calorie-normal phosphorus (LC-NP) and low calorie-high phosphorus (LC-HP).

Parameters	NC-NP	NC-HP	HC-NP	HC-HP	LC-NP	LC-HP
Triglycerides (mg/dL)	54.8 ± 11.1	34.9 ± 2.9	83.1 ± 11.4 *	39.6 ± 3.4^†^	22.3 ± 1.7 *	23.8 ± 2.3 *
Total cholesterol (mg/dL)	50.6 ± 7.7	35.3 ± 3.5	49.4 ± 6.1	37.9 ± 6.2	35.9 ± 3.3	52.8 ± 6.0^‡^
Glucose (mg/dL)	116.9 ± 6.1	94.1 ± 4.4 *	100.4 ± 6.1	103.6 ± 4.4	109.0 ± 19.6	74.2 ± 5.7^‡^
Leptin (ng/mL)	4.2 ± 0.5	3.1 ± 0.2	5.3 ± 0.8 *	4.1 ± 0.5	0.6 ± 0.2 *	0.6 ± 0.1 *
Adiponectin (ng/mL)	8.1 ± 1.0	11.3 ± 1.7	5.7 ± 0.5	7.7 ± 0.8	10.5 ± 0.6	16.6 ± 2.3 *^,‡^

Values are mean ± standard error (SE). * *p* < 0.05 vs. NC-NP, ^†^ *p* < 0.05 vs. HC-NP, ^‡^ *p* < 0.05 vs. LC-NP.
